# Bis(4-fluoro­anilinium) sulfate

**DOI:** 10.1107/S1600536811033137

**Published:** 2011-08-27

**Authors:** Hoong-Kun Fun, Suhana Arshad, Dinesha, Sandeep Laxmeshwar, G. K. Nagaraja

**Affiliations:** a X-ray Crystallography Unit, School of Physics, Universiti Sains Malaysia, 11800 USM, Penang, Malaysia; b Department of Chemistry, Mangalore University, Karnataka, India

## Abstract

In the crystal of the title molecular salt, 2C_6_H_7_FN^+^·SO_4_^2−^, the cations and anions are linked by N—H⋯O and C—H⋯O hydrogen bonds into sheets parallel to the *ab* plane. The crystal studied was found to be a racemic twin with a 0.50 (10):0.50 (10) domain ratio.

## Related literature

For related literature on phase transition dielectric materials, see: Fu *et al.* (2007 ▶, 2008 ▶, 2009 ▶); Fu & Xiong (2008 ▶). For hydrogen bonding studies, see: Zimmerman & Corbin (2000 ▶); Brunsveld *et al.* (2001 ▶); Desiraju (2002 ▶); Steiner (2002 ▶); Desiraju & Steiner (1999 ▶); Boutobba *et al.* (2010 ▶). For reference bond-length data, see: Allen *et al.* (1987 ▶). For a related crystal structure, see: Boutobba *et al.* (2010 ▶)
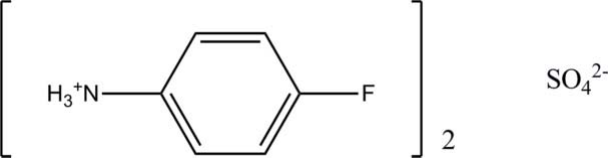


## Experimental

### 

#### Crystal data

2C_6_H_7_FN^+^·SO_4_^2−^
*M* *_r_* = 320.31
Orthorhombic, 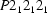

*a* = 6.2907 (5) Å
*b* = 7.4155 (6) Å
*c* = 30.168 (3) Å
*V* = 1407.3 (2) Å^3^
*Z* = 4
Mo *K*α radiation
μ = 0.27 mm^−1^
*T* = 296 K
0.58 × 0.12 × 0.07 mm

#### Data collection

Bruker SMART APEXII DUO CCD area-detector diffractometer
Absorption correction: multi-scan (*SADABS*; Bruker, 2009 ▶) *T*_min_ = 0.859, *T*_max_ = 0.982
31062 measured reflections
4292 independent reflections
3503 reflections with *I* > 2σ(*I*)
*R*_int_ = 0.044

#### Refinement

*R*[*F* ^2^ > 2σ(*F* ^2^)] = 0.038
*wR*(*F* ^2^) = 0.103
*S* = 0.98
4292 reflections
191 parameters
H-atom parameters constrained
Δρ_max_ = 0.18 e Å^−3^
Δρ_min_ = −0.39 e Å^−3^
Absolute structure: Flack (1983 ▶), 1794 Friedel pairs
Flack parameter: 0.50 (10)

### 

Data collection: *APEX2* (Bruker, 2009 ▶); cell refinement: *SAINT* (Bruker, 2009 ▶); data reduction: *SAINT*; program(s) used to solve structure: *SHELXTL* (Sheldrick, 2008 ▶); program(s) used to refine structure: *SHELXTL*; molecular graphics: *SHELXTL*; software used to prepare material for publication: *SHELXTL* and *PLATON* (Spek, 2009 ▶).

## Supplementary Material

Crystal structure: contains datablock(s) global, I. DOI: 10.1107/S1600536811033137/wn2446sup1.cif

Structure factors: contains datablock(s) I. DOI: 10.1107/S1600536811033137/wn2446Isup2.hkl

Supplementary material file. DOI: 10.1107/S1600536811033137/wn2446Isup3.cml

Additional supplementary materials:  crystallographic information; 3D view; checkCIF report

## Figures and Tables

**Table 1:** Hydrogen-bond geometry (Å, °)

*D*—H⋯*A*	*D*—H	H⋯*A*	*D*⋯*A*	*D*—H⋯*A*
N1—H3*N*1⋯O3	0.97	2.09	2.887 (3)	138
N2—H2*N*2⋯O3	0.85	1.90	2.741 (3)	168
N1—H1*N*1⋯O1^i^	0.86	1.92	2.761 (2)	162
N1—H2*N*1⋯O3^ii^	0.82	2.25	2.967 (2)	146
N1—H2*N*1⋯O4^ii^	0.82	2.55	3.151 (3)	131
N1—H3*N*1⋯O2^iii^	0.97	2.24	2.925 (2)	126
N2—H1*N*2⋯O4^iv^	0.94	1.78	2.7121 (18)	170
N2—H3*N*2⋯O2^v^	0.99	1.72	2.702 (2)	170
C11—H11*A*⋯O4^iii^	0.93	2.53	3.374 (3)	151

Symmetry codes: (i) 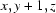; (ii) 
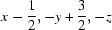
; (iii) 
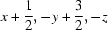
; (iv) 
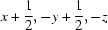
; (v) 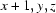.

**Fig. 1.**
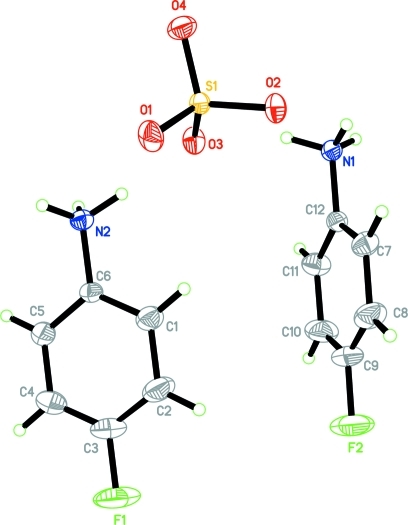
The molecular structure of the title compound with atom labels with 30% probability displacement ellipsoids. Hydrogen atoms are shown as spheres of arbitrary radius.

**Fig. 2.**
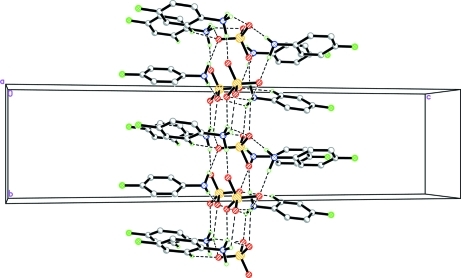
The crystal packing of the title compound. Dashed lines represent hydrogen bonds. H atoms not involved in the hydrogen bond interactions have been omitted for clarity.

## supplementary crystallographic information

### Comment

Amine salts have attracted much attention as phase transition dielectric materials for their application in memory storage (Fu *et al.* 2007; Fu & Xiong 2008; Fu *et al.* 2008; Fu *et al.* 2009). Hydrogen bonding is one of the most versatile non-covalent forces in supramolecular chemistry and crystal engineering (Zimmerman & Corbin, 2000; Brunsveld *et al.*, 2001; Desiraju, 2002). Therefore, in the past decades assessment of discrete hydrogen bonding patterns has received great attention (Steiner, 2002; Desiraju & Steiner, 1999; Boutobba *et al.*, 2010) because of their widespread occurrence in biological systems.

The asymmetric unit of the title compound (Fig 1), contains two crystallographically independent 4-fluoroanilinium cations and a sulfate anion. The bond lengths (Allen *et al.*, 1987) and angles are within normal ranges and comparable to those in a closely related crystal structure (Boutobba *et al.*, 2010).

The cations and anions are linked *via* intermolecular N1—H3N1···O3 and N2—H2N2···O3 hydrogen bonds (Table 1). In the crystal packing (Fig. 2), the intermolecular N1—H1N1···O1, N1—H2N1···O3, N1—H2N1···O4, N1—H3N1···O2, N2—H1N2···O4, N2—H3N2···O2 and C11—H11A···O4 hydrogen bonds (Table 1) link the molecules into sheets parallel to the *ab* plane.

### Experimental

To a solution of 4-fluoroaniline (10 mmol) in absolute ethanol was added sulfuric acid (5 drops) and the mixture refluxed for 4 h. After cooling the mixture to room temperature, a white solid appeared. This crude product was recrystallized from dimethylformamide to afford the desired product. *M.p*: 151–153°C.

### Refinement

N-bound H atoms were located from a difference Fourier map, fixed at their found location and refined using a riding model with *U*_iso_(H) = 1.5*U*_eq_(N) [N–H = 0.8198 to 0.9875 Å]. The remaining H atoms were positioned geometrically [C–H = 0.93 Å] and refined using a riding model with *U*_iso_(H) = 1.2 *U*_eq_(C). The studied crystal is an inversion twin, the refined ratio of twin components being 0.50 (10): 0.50 (10).

### Crystal data

**Table:**

2C_6_H_7_FN^+^·SO_4_^2^^−^	*F*(000) = 664
*M**_r_* = 320.31	*D*_x_ = 1.512 Mg m^−^^3^
Orthorhombic, *P*2_1_2_1_2_1_	Mo *K*α radiation, λ = 0.71073 Å
Hall symbol: P 2ac 2ab	Cell parameters from 5694 reflections
*a* = 6.2907 (5) Å	θ = 2.7–27.5°
*b* = 7.4155 (6) Å	µ = 0.27 mm^−^^1^
*c* = 30.168 (3) Å	*T* = 296 K
*V* = 1407.3 (2) Å^3^	Needle, colourless
*Z* = 4	0.58 × 0.12 × 0.07 mm

### Data collection

**Table:**

Bruker SMART APEXII DUO CCD area-detector diffractometer	4292 independent reflections
Radiation source: fine-focus sealed tube	3503 reflections with *I* > 2σ(*I*)
graphite	*R*_int_ = 0.044
φ and ω scans	θ_max_ = 30.6°, θ_min_ = 2.7°
Absorption correction: multi-scan (*SADABS*; Bruker, 2009)	*h* = −8→8
*T*_min_ = 0.859, *T*_max_ = 0.982	*k* = −10→10
31062 measured reflections	*l* = −43→43

### Refinement

**Table:**

Refinement on *F*^2^	Secondary atom site location: difference Fourier map
Least-squares matrix: full	Hydrogen site location: inferred from neighbouring sites
*R*[*F*^2^ > 2σ(*F*^2^)] = 0.038	H-atom parameters constrained
*wR*(*F*^2^) = 0.103	*w* = 1/[σ^2^(*F*_o_^2^) + (0.0492*P*)^2^ + 0.377*P*] where *P* = (*F*_o_^2^ + 2*F*_c_^2^)/3
*S* = 0.98	(Δ/σ)_max_ < 0.001
4292 reflections	Δρ_max_ = 0.18 e Å^−^^3^
191 parameters	Δρ_min_ = −0.39 e Å^−^^3^
0 restraints	Absolute structure: Flack (1983), 1794 Friedel pairs
Primary atom site location: structure-invariant direct methods	Flack parameter: 0.50 (10)

### Special details

**Table:**

Geometry. All e.s.d.'s (except the e.s.d. in the dihedral angle between two l.s. planes) are estimated using the full covariance matrix. The cell e.s.d.'s are taken into account individually in the estimation of e.s.d.'s in distances, angles and torsion angles; correlations between e.s.d.'s in cell parameters are only used when they are defined by crystal symmetry. An approximate (isotropic) treatment of cell e.s.d.'s is used for estimating e.s.d.'s involving l.s. planes.
Refinement. Refinement of *F*^2^ against ALL reflections. The weighted *R*-factor *wR* and goodness of fit *S* are based on *F*^2^, conventional *R*-factors *R* are based on *F*, with *F* set to zero for negative *F*^2^. The threshold expression of *F*^2^ > σ(*F*^2^) is used only for calculating *R*-factors(gt) *etc*. and is not relevant to the choice of reflections for refinement. *R*-factors based on *F*^2^ are statistically about twice as large as those based on *F*, and *R*- factors based on ALL data will be even larger.

### Fractional atomic coordinates and isotropic or equivalent isotropic displacement parameters (Å^2^)

**Table:**

	*x*	*y*	*z*	*U*_iso_*/*U*_eq_	
S1	−0.00432 (8)	0.47390 (4)	0.013801 (11)	0.02691 (9)	
F1	0.5089 (5)	0.3730 (3)	0.25139 (4)	0.1033 (6)	
F2	−0.0181 (4)	0.8372 (3)	0.22704 (4)	0.1096 (7)	
O1	−0.0040 (3)	0.29560 (14)	0.03416 (4)	0.0452 (3)	
O2	−0.2002 (2)	0.5713 (2)	0.02606 (5)	0.0372 (3)	
O3	0.1801 (2)	0.5795 (2)	0.02941 (5)	0.0381 (4)	
O4	0.0060 (3)	0.45980 (17)	−0.03476 (4)	0.0470 (3)	
N1	−0.0142 (3)	0.92624 (16)	0.04544 (4)	0.0309 (3)	
H1N1	0.0125	1.0369	0.0384	0.046*	
H2N1	−0.1252	0.8982	0.0330	0.046*	
H3N1	0.0935	0.8514	0.0315	0.046*	
N2	0.4960 (3)	0.37944 (17)	0.06828 (4)	0.0332 (3)	
H1N2	0.5163	0.2639	0.0563	0.050*	
H2N2	0.3856	0.4288	0.0573	0.050*	
H3N2	0.6038	0.4605	0.0553	0.050*	
C1	0.3283 (3)	0.4459 (4)	0.13955 (7)	0.0492 (5)	
H1A	0.2115	0.4926	0.1245	0.059*	
C2	0.3318 (4)	0.4435 (4)	0.18574 (7)	0.0639 (7)	
H2A	0.2179	0.4881	0.2020	0.077*	
C3	0.5047 (5)	0.3749 (3)	0.20625 (6)	0.0634 (6)	
C4	0.6765 (5)	0.3079 (4)	0.18406 (8)	0.0646 (7)	
H4A	0.7923	0.2611	0.1994	0.077*	
C5	0.6742 (4)	0.3113 (3)	0.13811 (7)	0.0487 (5)	
H5A	0.7901	0.2686	0.1221	0.058*	
C6	0.4990 (4)	0.37857 (19)	0.11639 (5)	0.0326 (3)	
C7	−0.1933 (3)	0.8307 (3)	0.11348 (6)	0.0434 (5)	
H7A	−0.3113	0.7982	0.0967	0.052*	
C8	−0.1934 (4)	0.8076 (4)	0.15908 (7)	0.0583 (6)	
H8A	−0.3101	0.7575	0.1734	0.070*	
C9	−0.0178 (5)	0.8602 (4)	0.18240 (6)	0.0636 (7)	
C10	0.1576 (4)	0.9328 (4)	0.16300 (8)	0.0671 (7)	
H10A	0.2739	0.9677	0.1800	0.081*	
C11	0.1593 (4)	0.9536 (3)	0.11737 (7)	0.0496 (5)	
H11A	0.2778	1.0015	0.1032	0.059*	
C12	−0.0157 (3)	0.9027 (2)	0.09331 (5)	0.0317 (3)	

### Atomic displacement parameters (Å^2^)

**Table:**

	*U*^11^	*U*^22^	*U*^33^	*U*^12^	*U*^13^	*U*^23^
S1	0.02748 (16)	0.02319 (15)	0.03006 (16)	−0.0005 (2)	0.0004 (2)	0.00064 (11)
F1	0.1150 (14)	0.1674 (18)	0.0274 (6)	−0.006 (2)	−0.0039 (9)	0.0034 (8)
F2	0.1171 (15)	0.1814 (19)	0.0301 (6)	0.013 (2)	0.0030 (10)	0.0157 (9)
O1	0.0549 (8)	0.0257 (5)	0.0550 (7)	0.0033 (10)	0.0022 (10)	0.0096 (5)
O2	0.0269 (6)	0.0333 (9)	0.0515 (8)	0.0032 (6)	0.0029 (6)	−0.0008 (7)
O3	0.0295 (6)	0.0324 (9)	0.0524 (8)	−0.0017 (6)	−0.0039 (6)	−0.0042 (7)
O4	0.0639 (8)	0.0467 (7)	0.0303 (6)	0.0010 (14)	0.0026 (8)	−0.0026 (5)
N1	0.0369 (7)	0.0261 (5)	0.0298 (6)	0.0007 (9)	0.0015 (7)	0.0008 (4)
N2	0.0321 (6)	0.0393 (6)	0.0283 (6)	−0.0015 (10)	0.0003 (9)	0.0001 (4)
C1	0.0423 (10)	0.0681 (15)	0.0373 (10)	0.0054 (11)	0.0034 (8)	−0.0018 (10)
C2	0.0595 (14)	0.095 (2)	0.0372 (11)	0.0064 (16)	0.0121 (10)	−0.0068 (13)
C3	0.0767 (16)	0.0857 (16)	0.0279 (8)	−0.008 (2)	−0.0039 (15)	0.0008 (8)
C4	0.0707 (17)	0.0807 (19)	0.0424 (12)	0.0137 (15)	−0.0198 (11)	0.0016 (12)
C5	0.0482 (12)	0.0584 (14)	0.0394 (10)	0.0137 (11)	−0.0061 (9)	−0.0058 (10)
C6	0.0364 (8)	0.0336 (7)	0.0279 (6)	−0.0029 (12)	−0.0022 (10)	−0.0012 (5)
C7	0.0436 (11)	0.0487 (12)	0.0380 (10)	−0.0036 (10)	0.0068 (8)	−0.0001 (9)
C8	0.0661 (16)	0.0679 (16)	0.0409 (11)	−0.0040 (14)	0.0180 (11)	0.0081 (11)
C9	0.0770 (18)	0.0866 (16)	0.0272 (8)	0.010 (2)	0.0032 (13)	0.0061 (9)
C10	0.0642 (15)	0.097 (2)	0.0400 (11)	−0.0012 (16)	−0.0178 (11)	−0.0017 (14)
C11	0.0458 (11)	0.0629 (14)	0.0399 (10)	−0.0086 (11)	−0.0051 (9)	0.0039 (10)
C12	0.0372 (9)	0.0281 (6)	0.0298 (7)	0.0017 (10)	0.0002 (9)	0.0012 (5)

### Geometric parameters (Å, °)

**Table:**

S1—O1	1.4579 (11)	C2—C3	1.350 (4)
S1—O4	1.4700 (11)	C2—H2A	0.9300
S1—O2	1.4755 (14)	C3—C4	1.365 (4)
S1—O3	1.4767 (14)	C4—C5	1.387 (3)
F1—C3	1.362 (2)	C4—H4A	0.9300
F2—C9	1.357 (2)	C5—C6	1.376 (3)
N1—C12	1.4548 (19)	C5—H5A	0.9300
N1—H1N1	0.8644	C7—C12	1.379 (3)
N1—H2N1	0.8198	C7—C8	1.386 (3)
N1—H3N1	0.9724	C7—H7A	0.9300
N2—C6	1.4513 (17)	C8—C9	1.366 (4)
N2—H1N2	0.9393	C8—H8A	0.9300
N2—H2N2	0.8524	C9—C10	1.360 (4)
N2—H3N2	0.9875	C10—C11	1.385 (3)
C1—C6	1.376 (3)	C10—H10A	0.9300
C1—C2	1.394 (3)	C11—C12	1.371 (3)
C1—H1A	0.9300	C11—H11A	0.9300
			
O1—S1—O4	110.81 (7)	C3—C4—C5	118.4 (2)
O1—S1—O2	109.84 (10)	C3—C4—H4A	120.8
O4—S1—O2	108.76 (10)	C5—C4—H4A	120.8
O1—S1—O3	110.21 (10)	C6—C5—C4	119.4 (2)
O4—S1—O3	108.71 (10)	C6—C5—H5A	120.3
O2—S1—O3	108.45 (7)	C4—C5—H5A	120.3
C12—N1—H1N1	111.1	C1—C6—C5	121.03 (16)
C12—N1—H2N1	114.8	C1—C6—N2	119.73 (19)
H1N1—N1—H2N1	107.0	C5—C6—N2	119.24 (19)
C12—N1—H3N1	111.5	C12—C7—C8	119.1 (2)
H1N1—N1—H3N1	107.5	C12—C7—H7A	120.5
H2N1—N1—H3N1	104.5	C8—C7—H7A	120.5
C6—N2—H1N2	112.3	C9—C8—C7	118.4 (2)
C6—N2—H2N2	113.7	C9—C8—H8A	120.8
H1N2—N2—H2N2	110.6	C7—C8—H8A	120.8
C6—N2—H3N2	113.0	F2—C9—C10	118.5 (3)
H1N2—N2—H3N2	108.0	F2—C9—C8	118.3 (3)
H2N2—N2—H3N2	98.3	C10—C9—C8	123.19 (19)
C6—C1—C2	119.4 (2)	C9—C10—C11	118.5 (2)
C6—C1—H1A	120.3	C9—C10—H10A	120.7
C2—C1—H1A	120.3	C11—C10—H10A	120.7
C3—C2—C1	118.4 (2)	C12—C11—C10	119.3 (2)
C3—C2—H2A	120.8	C12—C11—H11A	120.4
C1—C2—H2A	120.8	C10—C11—H11A	120.4
C2—C3—F1	118.5 (3)	C11—C12—C7	121.54 (17)
C2—C3—C4	123.4 (2)	C11—C12—N1	119.15 (19)
F1—C3—C4	118.1 (3)	C7—C12—N1	119.31 (18)
			
C6—C1—C2—C3	0.2 (4)	C12—C7—C8—C9	1.1 (4)
C1—C2—C3—F1	179.8 (2)	C7—C8—C9—F2	−179.9 (2)
C1—C2—C3—C4	0.1 (5)	C7—C8—C9—C10	−0.5 (4)
C2—C3—C4—C5	0.4 (5)	F2—C9—C10—C11	179.0 (3)
F1—C3—C4—C5	−179.3 (2)	C8—C9—C10—C11	−0.4 (5)
C3—C4—C5—C6	−1.1 (4)	C9—C10—C11—C12	0.8 (4)
C2—C1—C6—C5	−0.9 (4)	C10—C11—C12—C7	−0.2 (4)
C2—C1—C6—N2	179.6 (2)	C10—C11—C12—N1	179.4 (2)
C4—C5—C6—C1	1.4 (4)	C8—C7—C12—C11	−0.8 (3)
C4—C5—C6—N2	−179.1 (2)	C8—C7—C12—N1	179.62 (19)

### Hydrogen-bond geometry (Å, °)

**Table:**

*D*—H···*A*	*D*—H	H···*A*	*D*···*A*	*D*—H···*A*
N1—H3N1···O3	0.97	2.09	2.887 (3)	138
N2—H2N2···O3	0.85	1.90	2.741 (3)	168
N1—H1N1···O1^i^	0.86	1.92	2.761 (2)	162
N1—H2N1···O3^ii^	0.82	2.25	2.967 (2)	146.
N1—H2N1···O4^ii^	0.82	2.55	3.151 (3)	131.
N1—H3N1···O2^iii^	0.97	2.24	2.925 (2)	126.
N2—H1N2···O4^iv^	0.94	1.78	2.7121 (18)	170
N2—H3N2···O2^v^	0.99	1.72	2.702 (2)	170
C11—H11A···O4^iii^	0.93	2.53	3.374 (3)	151.

Symmetry codes: (i) *x*, *y*+1, *z*; (ii) *x*−1/2, −*y*+3/2, −*z*; (iii) *x*+1/2, −*y*+3/2, −*z*; (iv) *x*+1/2, −*y*+1/2, −*z*; (v) *x*+1, *y*, *z*.

## References

[bb1] Allen, F. H., Kennard, O., Watson, D. G., Brammer, L., Orpen, A. G. & Taylor, R. (1987). *J. Chem. Soc. Perkin Trans. 2*, pp. S1–19.

[bb2] Boutobba, Z., Direm, A. & Benali-Cherif, N. (2010). *Acta Cryst.* E**66**, o595–o596. 10.1107/S1600536810004782 PMC2983547 21580357

[bb3] Bruker (2009). *SADABS*, *APEX2* and *SAINT* Bruker AXS Inc., Madison, Wisconsin, USA.

[bb4] Brunsveld, L., Folmer, B. J. B., Meijer, E. W. & Sijbesma, R. P. (2001). *Chem. Rev.* **101**, 4071–4097. 10.1021/cr990125q 11740927

[bb5] Desiraju, G. R. (2002). *Acc. Chem. Res.* **35**, 565–573. 10.1021/ar010054t 12118996

[bb6] Desiraju, G. R. & Steiner, T. (1999). *The Weak Hydrogen Bond in Structural Chemistry and Biology*, p. 507. New York: Oxford University Press.

[bb7] Flack, H. D. (1983). *Acta Cryst.* A**39**, 876–881.

[bb8] Fu, D.-W., Ge, J.-Z., Dai, J., Ye, H.-Y. & Qu, Z.-R. (2009). *Inorg. Chem. Commun.* **12**, 994–997.

[bb9] Fu, D.-W., Song, Y.-M., Wang, G.-X., Ye, Q., Xiong, R.-G., Akutagawa, T., Nakamura, T., Chan, P. W. H. & Huang, S. P. D. (2007). *J. Am. Chem. Soc.* **129**, 5346–5347. 10.1021/ja0701816 17428055

[bb10] Fu, D.-W. & Xiong, R.-G. (2008). *Dalton Trans.* pp. 3946–3948. 10.1039/b806255b 18648695

[bb11] Fu, D.-W., Zhang, W. & Xiong, R.-G. (2008). *Cryst. Growth Des.* **8**, 3461–3464.

[bb12] Sheldrick, G. M. (2008). *Acta Cryst.* A**64**, 112–122. 10.1107/S0108767307043930 18156677

[bb13] Spek, A. L. (2009). *Acta Cryst.* D**65**, 148–155. 10.1107/S090744490804362X PMC2631630 19171970

[bb14] Steiner, T. (2002). *Angew. Chem. Int. Ed.* **41**, 48–76.

[bb15] Zimmerman, S. C. & Corbin, P. S. (2000). *Struct. Bond.* **96**, 63–94.

